# How can technology support ageing in place in healthy older adults? A systematic review

**DOI:** 10.1186/s40985-020-00143-4

**Published:** 2020-11-23

**Authors:** Aline Ollevier, Gabriel Aguiar, Marco Palomino, Ingeborg Sylvia Simpelaere

**Affiliations:** 1 VIVES University of Applied Sciences—Campus Bruges, Xaverianenstraat 10, Brugge, Belgium; 2 School of Engineering, Computing and Mathematics, University of Plymouth, Plymouth, UK; 3 Faculté de psychologie et sciences de l’éducation, Université Catholique de Louvain, Louvain-la-Neuve, Belgium

**Keywords:** Ageing in place, Older adult, Technology, Patient-centred

## Abstract

**Background:**

Ageing in place has recently gained visibility in healthcare policies and services. Technology has the potential to facilitate independence at home. The objective of this systematic review is to identify technologies that have been rigorously evaluated for supporting the ageing in place of healthy older adults. As well we explored the methods in engagement with technology in healthy older adults.

**Methods:**

Databases Pubmed, Scopus, PsycInfo and Cinahl were consulted for clinical controlled trials or randomised controlled trials between 2014 and 2019. Studies were included if they contained a technological intervention and focussed on supporting healthy older adults’ independent living. PRISMA guidelines and the risk of bias tool of the Cochrane Collaboration were applied.

**Results:**

The search identified 3662 articles of which only 7 made the final analysis. Through narrative analysis, technologies were categorised into three groups: accessible communication, emergency assistance and physical and mental well-being. Patient-centredness was extensively addressed by exploring how the participants engaged in the development and evaluation of the technology and how they were trained and monitored.

**Conclusions:**

Literature concerning technology to support ageing, based on controlled trials and research performed in authentic home situations, is scarce. Thus, there is a need to investigate the subject in depth. The use of a neurofeedback headband, an accessible computer system, a wristband with pedometer, a biofeedback device and an online video platform can bring added value to ageing in place for healthy older adults. A patient-centred approach for developing, implementing and evaluating technology benefits ageing in place.

**Supplementary Information:**

The online version contains supplementary material available at 10.1186/s40985-020-00143-4.

## Background

The world population continues to grow older. The number of older people is projected to grow more than 60% in the next 15 years. By 2030, there will be about 1 billion older people globally, which is equivalent to 12% of the total population [1]. Promoting healthy behaviours to prevent or reduce illness and disability among the ever expanding older population may neutralise the overwhelming demand for healthcare. Public health programmes will need to keep older people healthy for longer and postpone or avoid disability and dependency [1–3]. *Ageing in place* is a term widely used in the literature since 2010 [4]. While the term has various connotations, the WHO Centre for Health Development [5] defines ageing in place as “Meeting people’s desire and ability, through appropriate services and assistance, to continue to live relatively independently in the community, either in their current home or appropriate housing. Ageing in place aims to prevent or delay the trauma of moving to a facility such as a nursing home”. Ageing in place has also been referred to as “the ability to live safely, independently and comfortably in one’s own home and community, regardless of age, income or competence level” [6]. That makes ageing in place a shared responsibility of both the individual and the public authorities [3, 7].

To address the challenges posed by the ageing population, policies and public services should foster the focus on community life, rather than relying on institutionalised care [8]. Because technology is a potential resource to facilitate or improve ageing in place, it can contribute to the process of ageing independently at home, while improving health-related quality of life (HRQoL) [8–11]. Technologies enhancing ageing in place are known as information and communication technologies, health monitoring, assistive technologies, sensor technology, telemedicine, video games, medication reminders and internet of things (for example wearable devices) [8, 9, 12–20]. They are generally designed to monitor, support or improve activities of daily living, personal health or safety, mobility, communication and physical activities [8]. Digital technology can also help older people to emerge from social isolation, by strengthening their contact with the outside world, and their engagement in activities of their interest to boost their self-confidence [12].

Although the adoption of technology by healthy older adults who are ageing in place is a promising and theoretically well-founded idea [17, 21], there is a lack of high-quality studies in the subject [12]. The effectiveness of technology has been largely evaluated in other populations (for example, adults suffering from chronic conditions) [15].

Interacting and engaging with end-users is more common when designing digital technologies, this is also known as the principle of co-design [22, 23]. According to a recent systematic review, co-design lacks an unambiguous definition. The impact of co-design, when designing technologies to age in place, remains unclear because the focus relies on the variation and mixture of engagement. The exact engagement has a lot of variation and several blends are possible [24].

Therefore, the objective of this systematic review was to evaluate the effectiveness of technologies used at home by healthy older adults who are ageing in place. We aimed to identify relevant peer-reviewed and critical evaluated technological interventions for healthy older adults living independently at their home.

Of note, this review included only studies with the most rigorous evaluation and intervention designs, randomised controlled trials (RCT) and controlled clinical trials (CCT) (see the “Methods” section). From the reviewed interventions, we will also explore the engagement methods in participating to the interventions.

## Methods

Our systematic review was based on the Cochrane library manual for systematic reviews [25] and was conducted in accordance with the PRISMA guidelines [26]. There is no review protocol published. Systematic reviews on the field usually include RCT and CCT as inclusion criteria [27–30]. Even more, the Cochrane Handbook for Systematic Reviews of Interventions clearly defines RCT and CCT as main records for paper inclusion [25]. To define RCT and CCT as inclusion criteria allows authors to embrace in their review only papers that followed a strict method for reducing certain sources of bias when testing the effectiveness of new treatment, reducing spurious causality [31]. The applied methodology of RCT is often referred to as the gold standard for evaluating effectiveness and efficacy of healthcare interventions [32–34]. As a result, reviewers are capable of presenting papers that have accomplished the most reliable form of healthcare-outcome evidence in the hierarchy of evaluation that could influence healthcare practice [35].

### Inclusion and exclusion criteria

Studies published in English were included if they met the following criteria:

1. *Participants*. Independent living in the home of the participant’s choice (based on the definition of ageing in place [5], not in a residential care facility) and assumed healthy (no explicit conditions mentioned)
2. *Intervention*. Technological intervention (no disease-specific technology)
3. *Outcomes*. RCT or CCT

### Search strategy

We searched four databases: PubMed, Scopus, PsycInfo and Cinahl. We considered all publications from January 2014. Three concepts were used to determine the search terms: (a) participants met the definition of ageing in place, (b) technological intervention and (c) RCT or CCT.

We entered specific search terms for each concept, using keywords (e.g., MeSH) and free text words, combining with Boolean operators. The search keys were based on a combination of the following words: technological intervention, home adaptation, technology, tool, healthcare technology, health technology, eHealth, telehealth, mobile health, mHealth, telecommunication, telerehabilitation, self-help devices, assistive device, assistive technology, mobile devices, cloud computing, big data, artificial intelligence, age in place, community-dwelling, ageing in place, aging in place, independent living, randomized controlled trial, RCT, randomised controlled trial, controlled clinical trial, CCT, randomized control trial, randomised control trial, clinical control trial, controlled trial, control trial, randomized trial, randomized experiment and controlled experiment. Additionally, manual searches were conducted with Google Scholar, Cochrane and Epistemonikos or through citations and the snowball method. Full details of the search strategy and information sources can be found at the Supplemental Data—Searchkeys

### Study selection

Using the pre-defined inclusion criteria, all considered publications were first screened by title and abstract and then on the full texts by two independent researchers (first and second author). The results were compared and critically reviewed in an open discussion. In case of disagreement, a third researcher (last author) was consulted in order to reach a consensus. We obtained an inter-assessor reliability score of 87% as an agreement between the two independent reviewers.

### Quality appraisal

All included publications were subjected to a methodological quality assessment using the “Risk of bias tool” of the Cochrane Collaboration [36]. For each item of this tool, a summary with corresponding quotes from the first and second author was compiled. Following was the assignment of judgement in high, low or unclear risk. The results were discussed and the inter-assessor reliability score rose to 91%. Any disagreements were resolved by discussion with a third reviewer (last author). The quality appraisal can be found at Table 1 with full assessment available at the Supplemental data—Risk of Bias Tool for RCT.

**Table 1:**
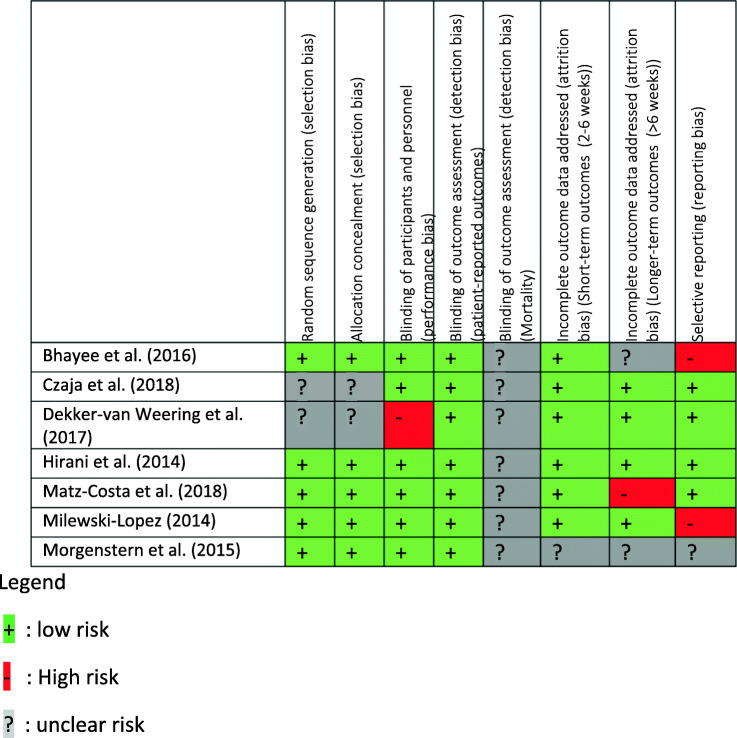
Risk of bias

### Data extraction

The following data were extracted and summarised in a table of evidence (Supplemental data—Table of Evidence): author and year, design, study objectives, population characteristics, sampling and sample size, intervention, outcome measures, and results.

### Data analysis

To make an inventory of all aspects of each intervention, our systematic review uses a narrative synthesis of the reviewed publications. We based the synthesis that classifies the technologies aimed at assisting older adults living independently on the process proposed by Ghapanchi and Aurum [37]. This process extracts terms and definitions to create an initial list of technologies and applications, after which the categories can be refined through further analysis.

## Results

**Fig. 1:**
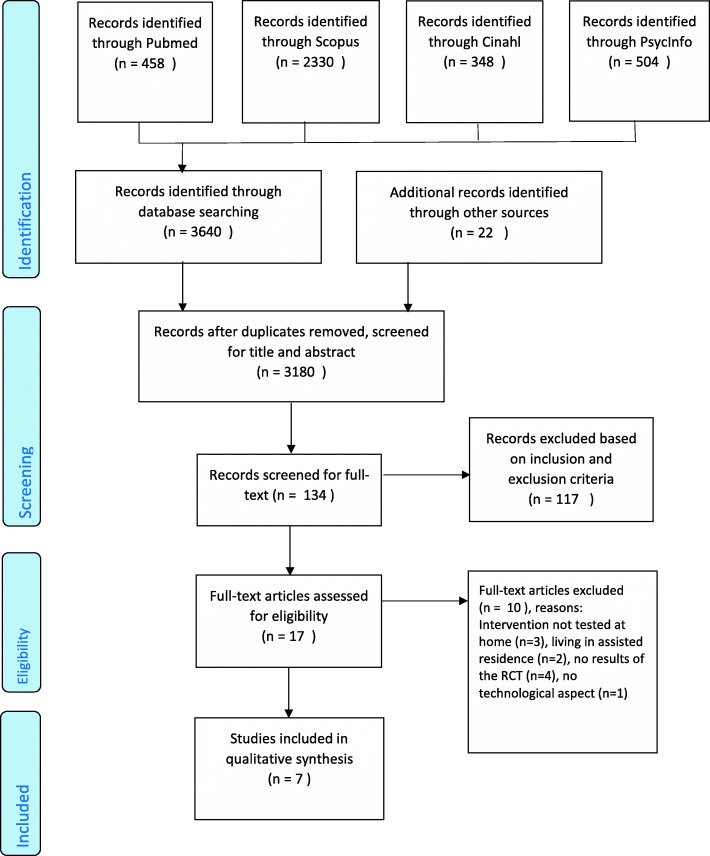
PRISMA flowchart

The PRISMA flowchart outlines the steps for the selection of the publications (Fig. 1).

The search yielded 3662 articles, of which only 7 were identified for the final analysis. All studies combined included 1904 participants (sample range, 30–1189). The studies were conducted in the USA [38–41], the UK [42, 43] and the Netherlands [44]. The average age for all included RCT’s was 68 years. Participants were mostly women. One study included only women [41], other studies had on average 67% women [38–40, 42, 44] and one study did not mention gender [43]. Various education levels were reported, an average of 30% participants had a high school education level or less [39, 41, 44], 50% had an education level between an associate and a bachelor's degree [40] and 64.8% had limited formal education [42]. Two publications reported insufficiently on the sample, leading to the absence of a clear insight into the basic characteristics of the included participants [38, 43].

Following the summary assessments of the risk of bias from the Cochrane Handbook for Systematic Reviews of Interventions, the selected studies present an overall low risk of bias (Table 1). This means that there is a low probability of having seriously altered results in the selected studies. As seen in Table 1, most of the studies present clear reports of the data collected and blinding of participants.

### Impact of the applied technologies—a narrative analysis

We identified several types of technologies that can be used in a home environment by independent living older adults in order to age in place. We took a large number of outputs and objectives into account. The narrative analysis of the results, based on the method of Ghapanchi and Aurum [37], was summarised in the following four categories.

#### Category 1: accessible communication

One publication examined the engagement and communication opportunities of a personal computer with dedicated software to access resources and information on the internet [39]. The results showed that the accessible computer system, aimed to improve communication and interaction, reduced loneliness and increased perceived social support and well-being compared to the control group. The results were reported at 6 months but not maintained at 12 months. The questionnaire for evaluating the acceptance of the technology showed positive feedback from the intervention group. Participants reported that the computer system was useful, improved their daily life, and made it easier to communicate with family and friends and to practice hobbies or play games. Researchers also observed an increase in efficiency, proficiency and comfort when using the computer at 6 and 12 months [39].

#### Category 2: emergency assistance

Two RCT’s evaluated similar emergency assistance technologies, including an alarm system with a pendant or bracelet [42] and an emergency assistance device connected to a wrist strap or necklace [41].

Using the pendant or bracelet system for 12 months had a positive impact on mental health, but other health-related, self-perceived outcomes, such as anxiety relief, were not achieved [42].

Participants who wore the wrist strap or necklace system for 90 days did not show any improvement in their HRQoL compared to the control group. This study only included female participants. Secondary outcomes such as changes in anxiety, depression and perceived isolation did not differ between the interventional group and the control group [41].

#### Category 3: physical well-being

A wristband with a pedometer [40] and online video platform for physical exercises [44] were identified as technologies within physical well-being. In the first study, the use of a pedometer, daily surveys, psycho-education or goal-setting workshops combined with active one-on-one mentoring over the phone, did not yield significant results compared to the control group. However, the number of daily steps increased by 11% compared to the baseline at week 4 for the intervention group (*p* < .05) and the findings persisted at week 8 (*p* < .01). Even though, there was a small decrease between week 4 and week 8. The number of daily steps in the control group decreased significantly compared to the baseline at week 4. This RCT had a small sample size and measured only the number of steps per day as physical activity [40].

The second study tested a 3-day physical training protocol for 12 weeks [44]. The protocol focused on strength and balance and was delivered via videos on a web page. Participants reported a good user experience and a high level of satisfaction with the study protocol, resulting in 68% adherence and a 20% drop-out rate at week 12. Although the sample size was small, the mental component of HRQoL showed significant improvements at week 12 compared to the control group [44].

#### Category 4: mental well-being

Two publications applied technology for improving participants’ mental well-being: a neurofeedback headband and electroencephalogram (EEG) with iPod-supported mindfulness training [38] and mental training on alertness with a biofeedback device [43].

For the first study, the outcomes were measured through self-reported questionnaires and objective measurements. Participants performed 10 min of mindfulness exercises every day for 6 weeks, using a headband to measure brain activity, EEG monitoring and an iPod. The results showed an improved reaction time in terms of attention and a reduction of somatic symptoms compared to the control group. Additional self-reported questionnaires did not show significant differences between the groups [38]. In the second study, a self-administered programme using Galvanic Skin Response to measure alertness showed a significant improvement in visual sustained attention, executive functioning and verbal fluency compared to a control group [43]. However, after the intervention, the intervention group showed no significant differences compared to the control group in terms of episodic memory, self-reported alertness or slips of memory and attention [43].

### Outcome measures

The subjective and objective outcome measures have been assessed in the various studies. These are different because the research goal was different each time. The outcome measures were collected at the start and had at least one measuring moment after the intervention. The majority of the studies contained self-perceived questionnaires. All outcome measures were summarised in the table of evidence.

### Engagement of the participants

Five out of seven included studies described interventions in which participants had to act actively, for example by performing exercises. These interventions cover three categories: accessible communication, physical well-being and mental well-being. The category regarding emergency assistance only required action when being in an emergency situation.

Therefore, it is remarkable that the same five studies extensively addressed the engagement of the participants by placing the participant central. Following is an overview in how the participants engaged in the included RCT’s.

In general, attitudes towards technology were positive, even when the participants had no or very limited computer experience. Older people are not only able to learn new technological skills, but they were very enthusiastic to work with the computer system when they were helped to do so [39]. How technology was presented and taught to the participants was also very important. Participants were positive about the information about the neurofeedback headband and EEG system regarding the expectations of participating [38]. Motivational strategies increased the extent to which participants adhered to the online video exercises in a self-management programme [44] with psycho-education, personal goal-setting and one-on-one peer mentoring in the use of the wristband with pedometer [40]. For the case of the use of an accessible computer system and an online video platform, participants welcomed the idea of technology implemented as a service, as opposed to technology as a stand-alone concept with enthusiasm and cooperation, provided they received appropriate guidance and assistance [39, 44]. Implementing a technology training session and a 2-day warm-up programme resulted in a high degree of adherence and engagement when using a wristband with pedometer [40]. Peer mentoring and careful follow-up over the phone received positive feedback as well [40]. In two studies, the design process reflected a patient-centred approach, older adults were involved in both the content creation for the training and instruction materials of the computer system [39] and thorough testing of the biofeedback device [43].

## Discussion

This review focussed on different types of technologies and their effectiveness for being used at home by healthy older adults to age in place. Furthermore, we explored the methods in engagement with technology in healthy older adults.

Together with the technology used in the home to enhance ageing in place, we chose only RCT or CCT for this review. These strict filters resulted in only 7 studies that have been implemented and evaluated in real-world contexts. Many technologies are used and tested in healthcare facilities; this review is unique since it focussed on technologies developed and evaluated at home. Evaluating with a CCT or RCT might not be easy to apply from a methodological point of view, but they represent the technology readiness prior to market access. We evaluated the quality of the included publications using the risk of bias tool and we obtaining satisfactory results. Blinding of outcome assessment regarding mortality could not be evaluated in all RCT due to the nature of all protocols. In addition, while there are ageing in place-related robotic solutions, voice assistants, virtual reality or exergames on the market, none of the studies focused on these high-tech innovations. This may be due to the small number of high-level RCT’s concerning the combinations of robotics or exergames and ageing in place. Consequently, this highlights the current need for evidence-based interventions to support healthcare decision-makers.

The results of all included papers cover a fairly young group of 1904 participants averaging 68 years of age, which was expected as this study focused on healthy older adults, not including disease-specific technology. The predominance of women in this study and the difficulty to involve male participants [40] was mentioned and both observations are in line with earlier research [45].

Most of the technologies showed significant markers for effectiveness with regard to ageing in place compared to the control group. The use of an accessible communication platform, an online video platform, a neurofeedback headband and a biofeedback showed significant results related to ageing in place in healthy older adults; however, non-significant differences were mentioned. Wearing a wristband with a pedometer increases the daily steps but other outcome measures could not be achieved. Only installing an emergency assistance device is not enough to enhance ageing in place in healthy older adults.

It was remarkable that many RCT contained patient-reported outcome assessments related to mental health and/or HRQoL and especially that these were often the only parameters where we observed a significant difference when compared to the control group. This may point to the significance of patient-reported subjective feelings in mental health and HRQoL and underscore its usefulness as an asset when implementing technologies for ageing in place. The technologies identified in this review are of similar categories as discussed in previous literature [8]. The literature also identifies technologies related to loneliness and social isolation [12]. Currently no instrument exists to assess the HRQoL for older adults who age in place. Such an instrument needs to be developed because any policy towards this growing group of people should be complemented by an evaluation [46].

Besides, even though some positive results were observed in the studies presented, in some specific interventions [40, 43], positive healthcare outcomes faded away with time. This could be attributed to the novelty effect of technology. When presented a new technology, research participants present high levels of engagement, but over time, the users start losing interest due to different aspects. This has been evidenced and studied by other studies that explore how to motivate users for long-term use of assistive technology [47–50]. This is an important point that requires further research and attention from the research community. Even if we are designing a technology that could improve the health and well-being of our user, without exploring methods to increase the intrinsic motivation, we will not achieve long-term adherence.

This review adds to the literature by underlining the positive attitude older adults have towards technology. A patient-centred approach in this context means education, training, guidance, a close follow-up and including the participant in the development and evaluation of the technology. This participation contributes to the principles of co-design. Our results regarding the importance of patient-centred care reinforce earlier research [17, 23]. Because our findings are relevant within their context, we consider them to be viable and effective, although this review was not an evaluation of patient-centredness.

### Strengths

This study raises awareness of technologies that have been used and evaluated under strict testing methods (RCT and CCT). As a result, the technologies and therapies suggested in this review will be more likely to have the impact described by their respective authors. Furthermore, this literature review followed a strict methodology that allowed the researchers to reduce bias and spurious causality.

### Limitations

As explained in the methods section, this review only presents studies that worked exclusively with healthy older adults without special conditions or impairments. We follow this path to identified validated technologies that could help this population group that is currently on the rise [1–3]. However, this could have limited our sample of studies, as it was evidenced by this review that an important number of studies focused on vulnerable groups (e.g., dementia, chronic obstructive pulmonary disease, heart conditions). However, at the same time, this also raises awareness of the need for further research to explore the challenges and technology developments for seniors without special conditions, affected by the normal conditions of ageing. More publications could have been included if we had consulted more databases and had been less specific in the design choice, for example by including laboratory research. We believe that valuable publications with good practices are available, but for this research, we only used studies with a CCT or RCT design. Another limitation was not using the GRADE assessment to provide levels of recommendations. Because of the wide range of purposes the technology envisions, we did not consider this necessary.

## Conclusion

Research concerning technology to age in place performed in an authentic situation, supported with a randomised controlled trial, is rare. More research on long-term effectiveness is needed.

The identified technologies that support ageing in place focus on accessible communication, emergency assistance, physical well-being and mental well-being. When studying their effectiveness, we see that the use of a neurofeedback headband and EEG system, an accessible computer system, a wristband with pedometer, a biofeedback device and an online video platform can bring added value to ageing in place. Patient-centredness is crucial when developing and evaluating technology, and when integrating technology at home, it demands training, guidance and close follow-up of the participants.

## Supplementary Information

**Additional file 1.** Supplemental Data.

## Data Availability

All data generated or analysed during this study are included in this published article [and its supplementary information files].

## Abbreviations

RCT: Randomised controlled trials
CCT: Controlled clinical trials
HRQoL: Health-related quality of life
EEG: Electroencephalogram
