# Supplementary material for: How can technology support ageing in place in healthy older adults? A systematic review
Source: Public Health Rev. 2020 Nov 23;41:26. doi: 10.1186/s40985-020-00143-4 (PMC7684947; doi:10.1186/s40985-020-00143-4)
Supplement: Supplementary file 1 — Additional file 1. Supplemental Data. [file 40985_2020_143_MOESM1_ESM.docx]

| **Supplemental Data - searchkeys** | |
| --- | --- |
| Medline | ("home adaptation" OR technology OR tool OR ehealth OR "Self-Help Devices"[Mesh] OR "Telecommunications"[Mesh] OR "Artificial Intelligence"[Mesh]) AND (“age in place” OR “ageing in place” OR "Independent Living"[Mesh]) Filter: >2014 |
| Scopus | ( "technological intervention"  OR  "home adaptation"  OR  technology  OR  tool  OR  "healthcare technology"  OR  "health technology"  OR  ehealth  OR  telehealth  OR  "mobile health"  OR  mhealth  OR  telecommunication  OR  telerehabilitation  OR  "self-help devices"  OR  "assistive device"  OR  "assistive technology"  OR  "mobile devices"  OR  "cloud computing"  OR  "big data"  OR  "artificial intelligence" )  AND  ( "age in place"  OR  "ageing in place"  OR  "aging in place" )  Filter: >2014 |
| Cinahl | (“technological intervention” OR “home adaptation” OR technology OR tool OR “healthcare technology” OR “health technology” OR eHealth OR telehealth OR “mobile health” OR mHealth OR telecommunication OR telerehabilitation OR “self-help devices” OR “assistive device” OR “assistive technology” OR “mobile devices” OR “cloud computing” OR “big data” OR “artificial intelligence”) AND (“age in place” OR “community dwelling” OR “ageing in place” OR “aging in place” OR “independent living”)  Filter: >2014 |
| PsyInfo | (“technological intervention” OR “home adaptation” OR technology OR tool OR “healthcare technology” OR “health technology” OR eHealth OR telehealth OR “mobile health” OR mHealth OR telecommunication OR telerehabilitation OR “self-help devices” OR “assistive device” OR “assistive technology” OR “mobile devices” OR “cloud computing” OR “big data” OR “artificial intelligence”) AND (“age in place” OR “community dwelling” OR “ageing in place” OR “aging in place” OR “independent living”)  Filter: >2014 |
| Filter on design | Applying filter RCT in EndNotel itself (searches title and abstract): randomized controlled trial OR RCT OR randomised controlled trial OR controlled clinical trial OR CCT OR randomized control trial OR randomised control trial OR clinical control trial OR controlled trial OR control trial OR Randomized trial OR randomized experiment OR controlled experiment |

| **Supplemental data –** Table of evidence | | | | | | | |
| --- | --- | --- | --- | --- | --- | --- | --- |
| **Author & year** | **Design** | **Study objectives** | **Population characteristics** | **Sampling and sample size** | **Intervention** | **Outcome measures** | **Results** |
| **Bhayee et al 2016** | RCT | The benefit of N-tsMT for attention and or wellbeing in a ecologically-valid research | Healthy adult, community dwelling under moderate or high stress | Canada  N:43  Intervention: 20 (2 dropout)  Control: 23 (6 dropout) | Intervention: a brain sensing headband, wireless EEG headset, mobile device to wireless transform data, calibration and an Ipod for guided meditation instructions through auditory cues. Daily training for 10minutes for 6 weeks  Control: free, online and at high school level algebra class. Daily training for 10minutes for 6 weeks. | Primary assessment   - Stroop task for attention - Brief Symptom Inventory (BSI) for psychological distress   Exploratory measures   - D2task for concentrative attention - Digit span task for working memory - Freiburg Mindfulness Inventory (FMI) for mindfulness - Positive and Negative Affective schedule (PANAS) for emotional state - WHOQOL-BREF for mental and physical health | Intervention group significantly improved in overall reaction time on the stroop task when compared to the control group (Z=3.29, p<.001, r=.65). The somatic subscale of the BSI showed significant interaction between intervention and control group, meaning reduced somatic symptoms compared to the control (Z=2.81, p=.004, r=.55). Attention and well-being improvements were correlated, and effects were greatest for the most neurotic participants (r(11)=-.70, p=.007).  The exploratory measures revealed no significant interactions within groups SD (.03-.31). The interventions was associated with greater body awareness (t(36) =2.03, p<.05) and calm (t(36) =2.16, p = .04), and initially greater effort that later converged with effort in the control condition (t(1047)=-2.00, p=.046). |
| **Czaja et al. (2017)** | RCT | Evaluated the impact of the personal reminder information and social mangagement (PRISM) system | Individuals aged 65 or older living alone in independent housing, who speak English and are at risk for social isolation | US  N: 300  Intervention: 150 (45dropouts)  Control: 150 (31dropouts) | Intervention: received a pc, the PRISM software application and a printer. The personal reminder information and social management (PRISM), is a software application and a support system with training and instructional support. Easy access to resources and communication and carefully preselected features. Content: vetted links, annotated resource guide, a dynamic classroom feature, a calender, a photo feature, e mail, games and online help. The classroom feature was dynamic and updated monthly with an information on a new topic and included three tables.  Control: received a notebook with similar content to that within PRISM | Assessed at baseline, 6, 12, 18 months   - Friendship scale for social isolation - Loneliness scale for loneliness - Interpersonal support evaluation list for social support - Lubben social network index for social network size - Quality of life scale for perceptions of QoL - Perceived vulnerablility scale for the perceived vulnerability - MOS 36item short-form health survey   Secondary outcomes included changes in attitudes toward technology and computer proficiency and technology acceptance questionnaire. | Significantly less loneliness(b=1.72, p<.04, effect size .17) and increased perceived social support (b=-1.96, p<0.04, effectsize: 0.28) and well-being (b=-4.86, p<0.02,effect size =.27) at 6months within interventional group versus control. Group differences were not maintained at 12 months, but those in the PRISM condition still showed improvements from baseline. All with small effectsizes (range: 0.17 – 0.28).  There was also an increase in computer efficacy (b = −1.29; p < .001; effect size = 0.41; 95% CI = −2.01 to −0.57) and 12 months (b = −0.94; p < .02; effect size = 0.30; 95% CI = −1.67 to −0.22), proficiency at 6 months (b = −6.37, p < .001; effect size = 1.11; 95% CI = −7.39 to −5.35) and 12 months (b = −7.06, p < .001; effect size = 1.23; 95% CI = −8.08 to −6.03), and comfort at 6 months (b = −1.68; p < .001; effect size = 0.39; 95% CI = −2.57 to −0.78) and 12 months (b = −2.32; p < .001; effect size = 0.53; 95% CI = −3.22 to −1.41). |
| **Dekker-van Weering M et al. (2017)** | RCT | 1.Investigate the use and user experience of an Information Communication Technology-supported home exercise program for independent use to pre-frail older adults 2.Explore whether the program improved quality of life and health status compared to a control group | Pre-frail older adults (65–75 years) living independently at home | The Netherlands  N = 37  Intervention: 15 (1 dropout)  Control: 21  Sample size calculation:  A power calculation based on t-test testing shows that at least 40 patients per group are needed | Intervention: technology-supported self-management exercise program, which participants can perform in their home setting, through log in on a website.  The program consists of a home-based exercise training that enables participants to train for 3 months (3x/week 30min. for 12 weeks). Participants can continue training after program completion. The exercise program is based on the OEP (Otago Excercise Program), which is an individually tailored fall prevention program used worldwide for muscle strengthening and balance-training exercises with increasing intensity. The exercises are functional and closely related to daily activities (e.g., standing up from a chair) and have three categories: balance, strength, and flexibility. The program consists of 17 exercises each time. Each training session starts with a warming-up of five exercises and ends with a cooling down with four exercises. Each training sessions covers the three categories. Video and step-by-step spoken and written instruction guide the participants through the exercise.  Control group: no access to the exercise program and received care as usual with no particular attention, referral, or treatment. | Primary outcome:  At t1 (12 weeks) intervention group:   1. The use of the intervention by logging data on the portal, defined in frequency and duration of log in. 2. Adherence to the program by calculating completion of the training session as indicated by watching the exercise videos. 3. User experience with the System Usability Scale (SUS)   Secondary outcome:  At t0 and t1, both intervention and control:  QoL was measured by SF-12v1 (12-item Short Form questionnaire version 1), Health status was measured by EQ-5D-3L. | Primary outcome:   1. Participants rated the intervention with an 8.5. Completion of the 12week exercise protocol was 80%. Duration of the training was an average of 2.2 times each week. The mean duration of login for each exercise session was 24 min. 2. The adherence to the 3-day exercise protocol was 68%. 3. The average score on the SUS was 84.2, almost reaching an excellent score.   Secondary outcome (cfr. Table 2):  The Mental Component Scale of the SF12 was significantly higher in the intervention group compared to the control group (p=0.016). A trend was seen in the change over time in the health status between groups. No significant differences between groups for the health status and the physical component of Quality of Life |
| **Hirani et al. (2014)** | Cluster-RCT | What is the effect of home-based telecare (TC) on health-related quality of life (HRQoL), anxiety and depressive symptoms over 12 months in patients receiving social care? | Individuals aged 18 or older. | UK  N= 1189  Intervention group = 550  Control group = 639  Power calculation: The required minimum sample size was 550. | Intervention group: for 12 months the Tunstall Lifeline Connect or Connect+ base unit and pendant/bracelet alarm alongside any number of up to 27 peripheral devices. Following any alert, monitoring centre staff attempted to make contact with the individual, via the base unit or telephone, and if further assistance was required contact was made with an identified carer or emergency services, as appropriate.  Control group: usual health and social care for the 12-month duration of the trial. Some control participants received a pendant/bracelet alarm as this was current UC practice. | All questionnaires were self-completed by the participant at baseline, short-term (4months) and longterm (12 months).   - Short Form 12-item Survey (SF-12) for HRQoL - EQ-5D York-Tariff summary index for overall health - ICECAP-O index for capability for older people - Brief STAI for anxiety - CESD-10 for depressive symptoms | The results show that telecare increases the SF12 mental component score in between groups (p=0.017), with small effect size.  The health status is reduced from 4 months to 12 months (p =0.002) and depressive symptoms increased from 4 to 8 months (p=0.032). No effect was found on anxiety. |
| **Matz-Costa et al. (2018)** | RCT | To evaluate the feasibility and outcomes of the Engaged4Life program, into their everyday lives | Individuals >65, relatively inactive  The majority of participants were women (80%), non-Hispanic White (92%), retired (68%), and married (52%). | US, Massachusetts  N = 30  Intervention = 15 (3dropouts)  Control = 15 (2 dropouts) | Engaged4Life program, an intervention to encourage inactive community-dwelling older adults to embed physical activity, cognitive activity, and social interaction into their everyday lives in contexts that are personally meaningful and natural for them. Duration of study: 8 weeks.  Intervention group: technology-assisted selfmonitoring of daily activity; daily tabletbased surveys, psychoeducation + goal-setting (via a 3-hour workshop), and (c) one-on-one peer mentoring (via phone 2×/ week for 2.5 weeks) to support goal implementation.  Self-monitoring-only control group: received only technology-assisted self-monitoring of activity engagement. | Primary outcome:  FITBIT® pedometers for daily step count and physical activity  Secondary outcomes:  Daily tabletbased self-repot survey for cognitive activity, quantity and quality of social interactions, and perceptions of meaning. | This pilot trial was not powered to demonstrate significant differences between groups, daily steps increased by 431 (11% increase) from baseline to Week 4 for the intervention (p < .05), but decreased by 458 for the control, for a net difference of 889 steps (p= < 0.05). Findings were sustained at Week 8 (p= <0 .01). |
| **Milewki-Lopez 2014** | RCT | What’s the efficicacy of the Alertness Training for Focused Living programme in improving aspects of memory, attention, and executive functioning in a group of older adults? | Self-reported difficulties with everyday memory and attention, overall good health, English speaking, living at home independently and without any neurological or psychiatric illness | Dublin  N=40  No information on sample or adherence | Interventional group: alertness training for focused living had 4 components   - Awareness on alertness through a guidebook - Self alert technique, from week 2, taught through the guidebook and tutorial cd - Biofeedback device, from week 3, in the form of a cushion for recording and displaying galvanic skin response (GSR) in real-time. - Alertness goals for applying the self alert technique during chosen activities   Control group: Attention education programme through a guidebook focusing on general aspects of attentional processes, alertness of the body, alertness and performance and attention and daily living. | Pre-post and 1month follow up, objective measures follow up for 3 and 6months  Subjective self-report assessments   - Attention-Related Cognitive Errors Scale (ARCES) for attention slips and absentmindedness - Memory Failures Scale (MFS) for minor memory failures - Toronto Hospital Alertness Test (THAT) for perceived alertness - Hospital Anxiety and Depression Scale (HADS) for psychological stress.   Objective assessments   - Rivermead Behavioral Memory Test (RBMT) for immediate and delayed story recall - Word list immediate and delayed recall for words recall - Vigilant Auditory Attention Task (VAAT) for visual sustained attention - Category Fluency for executive functioning | For the subjective assessments there was no significant difference between intervention and control during prepost and 1month follow up except for the HADS at 1month follow up, F(1, 34) = 6.11, p = 0.02, η2 = 0.15, meaning that trainees reported a significantly higher level of psychological stress than controls.  For the objective assessments there was a statistically significant effect of training, with small effect size, η2 = 0.19, on VAAT ERCs, F(1, 26) = 6.26, p = 0.02. Trainees made significantly fewer errors than controls in the post-training assessment, F(1, 32) = 5.81, p = 0.02, η2 = 0.15. While the difference between trainees and controls was not statistically significant during the 1-month, F(1,32) < 1, and 3-month follow-ups, F(1, 29) = 2.08, p = 0.16, they were again found to make significantly fewer errors than controls during the 6-month follow-up assessment, F(1, 28) = 6.17, p = 0.02, η2 = 0.18.  There was also a significant effect of training on Category Fluency. There was a statistically significant effect of Group during the post-training assessment session, F(1, 34) = 12.94, p = 0.001, η2 = 0.28, with trainees recalling a significantly higher number of items than controls, relative to baseline scores. The effect of Group was not statistically significant across all post-training assessments, F(1, 31) = 1.43, p = 0.24, however, indicating that this improvement was not maintained across follow-up sessions (see Table 2). The effect of training was not statistically significant for  Immediate or Delayed Story Recall, Immediate or Delayed Word Recall or EROs and RTCov on the VAAT. |
| **Morgenstern et al. (2015)** | RCT | To examine the benefit of wearing medical alert devices to activate emergency medical systems for elderly women living alone. | Women aged >60 with minimum 1 stroke risk factor  On average, the treatment group was older, reported lower prevalence of high cholesterol, and was less likely to complete follow-up. | Us, Michigan  N= 265  Intervention: 133 (21 dropouts)  Control: 132 (10 dropouts) | Intervention: received the medical assistance device Huron Valley Ambulance (HVA), speakerphone Visonic Amber Select and instructions for its use along with the call button device Cisonic MCT-212, for 90 days. If a person pushed the call button, it would communicate a singal to HVA.  Control: no detailed information | The primary outcome:   - The HRQOL for quality of life.   The secondary outcomes   - HADS for anxiety and depression - Modified version of the perceived isolation index for social connectedness - Name stroke warning signs for stroke knowledge | This study did not establish improvement in HRQOL among women who wore the device compared with those that did not, nor the feasibility of a trial to study the efficacy of medical alert devices in elderly women. There was a non-significant smaller loss of healthy days in the past month in the intervention group (0.46) compared with the control group (2.23), (p=0.213). Similarly, the secondary outcomes of changes in anxiety, depression and changes in perceived isolation did not differ by treatment and control groups. |

Supplemental Data – Risk of Bias Tool for RCT

| **Bhayee (2016)** | | |
| --- | --- | --- |
| Random sequence generation (selection bias) |  | Quote: “randomization was performed using the random number generator function in the MATLAB programming environment for subblocks of 4 participants in each condition”  Comment: |
| Allocation concealment (selection bias) |  | Quote: “conducted by the principal investigator without participant contact” |
| Blinding of participants and personnel (performance bias) |  | Quote: “Participants were blind to experimental condition while completing the baseline assessment battery” |
| Blinding of outcome assessment (detection bias) (patient-reported outcomes) |  | Comment: no blinding, outcome measurement is not likely to be influenced by lack of blinding |
| Incomplete outcome data addressed (attrition bias) (Short-term outcomes  (2-6 weeks)) |  | Quote:  Comment: Missing data not mentioned ,all outcomes are addressed |
| Incomplete outcome data addressed (attrition bias) (Longer-term outcomes  (>6 weeks)) |  | Comment: only 6 weeks follow-up |
| Selective reporting (reporting bias) |  | Comment: Unsufficient reporting concerning the study sample, not enough basic characteristics mentioned or referred to. |

| **Czaja et al. (2018)** | | |
| --- | --- | --- |
| Random sequence generation (selection bias) |  | Quote: “participants were randomly assigned to either the intervention condition or a control condition”  Comment: no randomization method described |
| Allocation concealment (selection bias) |  | Comment: No allocation concealment method mentioned |
| Blinding of participants and personnel (performance bias) |  | Quote: “An assessor blinded to treatment condition, administered the primary outcome measures of the assessment battery at 6 and 12 months via a telephone interview” |
| Blinding of outcome assessment (detection bias) (patient-reported outcomes) |  | Comment: no blinding, outcome measurement is not likely to be influenced by lack of blinding |
| Incomplete outcome data addressed (attrition bias) (Short-term outcomes  (2-6 weeks)) |  | Quote: Missing data were handled using restricted  maximum likelihood. Low dropout  Comment: all outcomes are addressed |
| Incomplete outcome data addressed (attrition bias) (Longer-term outcomes  (>6 weeks)) |  | Missing data were handled using restricted  maximum likelihood. Low dropout  Comment: all outcomes are addressed |
| Selective reporting (reporting bias) |  | Quote: "The Institutional Review Boards at the three sites approved the study protocol”  Comment: Protocol is available and outputs mentioned there are the ones presented in this study |

| **Dekker-van Weering M et al. (2017)** | | |
| --- | --- | --- |
| Random sequence generation (selection bias) |  | Quote: “Randomly assigned to a control group or an intervention group.”  Comment: no randomization method described |
| Allocation concealment (selection bias) |  | Quote: “flow diagram shows allocation of participants to the control or intervention group”  Comment: participants were recruited through their general practitioner, no clear method described |
| Blinding of participants and personnel (performance bias) |  | Quote: “testers were not blinded to group assignment for ease of recruitment and to accommodate for time constrains”  Comment: no blinding of participants mentioned |
| Blinding of outcome assessment (detection bias) (patient-reported outcomes) |  | Comment: outcome measurement is not likely to be influenced by the lack of blinding |
| Incomplete outcome data addressed (attrition bias) (Short-term outcomes  (2-6 weeks)) |  | Quote: “The adherence to this protocol was 68%, dropout rate 20%”.  Comment: Unclear how missing data was handled |
| Incomplete outcome data addressed (attrition bias) (Longer-term outcomes  (>6 weeks)) |  | Comment: It seems that the outcomes of this study were reported for the 3 months. Low amount of missing data However: Unclear how missing data was handled. |
| Selective reporting (reporting bias) |  | Quote: “The study was part of the European FP7 project PERSSILAA. This study was the first wave of a cohort multiple randomized controlled trail (Jansen-Kosterink et al., submitted).”  Comment: It seems that not all outcomes of the complete study were reported in this article; however, those outcomes related to the research question for this study were reported in this study |

| **Hirani et al. (2014)** | | |
| --- | --- | --- |
| Random sequence generation (selection bias) |  | Quote: “Consenting practices were allocated to the TC (n = 101) or usual care (UC) (n = 103) group using a centrally administered minimization algorithm devised to ensure groups of practices and were similar in terms of size, deprivation index, proportion of White patients and the presence of social care needs.” “enrolled and randomized into the intervention and control groups” Comment: minimization |
| Allocation concealment (selection bias) |  | Quote: “allocation to trial-arm was conducted using cluster randomization, based on participants’ registration with a particular general practice (GP). |
| Blinding of participants and personnel (performance bias) |  | Quote: “Neither participants nor assessors could be blinded to trial-arm allocation, due to the nature of the intervention”  Comment: blinding could not be performed because of the intervention, outcome unlikely to be influenced |
| Blinding of outcome assessment (detection bias) (patient-reported outcomes) |  | Quote: “Neither participants nor assessors could be blinded to trial-arm allocation, due to the nature of the intervention”  Comment: No blinding of outcome assessment, but the review authors judge that the outcome measurement is not likely to be influence by lack of blinding. |
| Incomplete outcome data addressed (attrition bias) (Short-term outcomes  (2-6 weeks)) |  | Quote: “A potential limitation is that rates of loss to follow-up at ST were substantial and were slightly different for the intervention and control groups.” “missing data were imputed using the SPSS MCMC function within each administration  Comment: It seems that all of the study’s outcomes have been reported. There is an explanation for missing responses and for withdrawal. |
| Incomplete outcome data addressed (attrition bias) (Longer-term outcomes  (>6 weeks)) |  | Quote: “ Missing data rates at longterm were low and were imputed using SPSS MCMC function within each administration”  Comment: It seems that all of the study’s outcomes have been reported. There is an explanation for missing responses and for withdrawal. |
| Selective reporting (reporting bias) |  | Quote: International Standard Randomised Controlled Trial Number Register ISRCTN43002091” “study protocol was approved by the Liverpool Research Ethics Committee (REF: 08/H1005/4)  Comment: all expected outcomes are reported |

| **Matz-Costa et al. (2018)** | | |
| --- | --- | --- |
| Random sequence generation (selection bias) |  | Quote: “The Tripod Random Allocation Software was used to generate the random allocation sequence using a 1:1 ratio.”  Comment: Investigators describe a random component in the sequence generation. |
| Allocation concealment (selection bias) |  | Quote: “Intervention assignments were placed in sealed, non-translucent envelopes by the first author and screeners chose the envelopes, in sequence, to assign eligible individuals to an intervention group”. This was done to conceal the random allocation sequence to the screeners until interventions were assigned”  Comment: clear description in the text |
| Blinding of participants and personnel (performance bias) |  | Quote: “Participants were blinded to treatment group.”  Comment: participants received the device or not, randomization was clear selecting control and intervention participants. |
| Blinding of outcome assessment (detection bias) (patient-reported outcomes) |  | Comment: outcome measurement is not likely to be influenced by the lack of blinding |
| Incomplete outcome data addressed (attrition bias) (Short-term outcomes  (2-6 weeks)) |  | Quote: “LME accommodates missing data for participants with missing data points, thus models could use data from the subset of the sample who were lost to follow-up”  Comment: Missing data have been imputed using appropriate methods. |
| Incomplete outcome data addressed (attrition bias) (Longer-term outcomes  (>6 weeks)) |  | Quote: “only measurements included till 2-month”  “and we were not able to follow participants beyond the 2-month period”  Comment: all outcome reported until 2 months. No follow up after 8 weeks, only primary outcomes reported at 2 months, no secondary |
| Selective reporting (reporting bias) |  | The trial  was registered retrospectively with clinicaltrials.gov (NCT03337204, date:  November 8, 2017) and approved by the Boston College IRB (protocol #15.063.04) and the Massachusetts Executive Office of Elder Affairs’ ElderRights Review Committee. |

| **Milewski-Lopez 2014** | | |
| --- | --- | --- |
| Random sequence generation (selection bias) |  | Quote: “randomly assigned to the Trainee or Control group using a computer algoritm minimization.”  Comment: |
| Allocation concealment (selection bias) |  | Quote: “the procedure ensured the groups were matched for age and gender”  Comment: |
| Blinding of participants and personnel (performance bias) |  | Quote: ‘research assistant was blind to group allocation”  Comment: |
| Blinding of outcome assessment (detection bias) (patient-reported outcomes) |  | Comment: self-administred tests not applicable |
| Incomplete outcome data addressed (attrition bias) (Short-term outcomes  (2-6 weeks)) |  | Comment: all outcomes are well reported |
| Incomplete outcome data addressed (attrition bias) (Longer-term outcomes  (>6 weeks)) |  | Comment: clearly motivated why not all data is reported |
| Selective reporting (reporting bias) |  | Comment: no information on adherence, not enough reporting on the basic characteristics o the sample |

| **Morgenstern et al. (2015)** | | |
| --- | --- | --- |
| Random sequence generation (selection bias) |  | Quote: “Eligible subjects were randomized to either the medical assistance device or control in a block design with block sizes of 4 and 6.”  Comment: clear method described |
| Allocation concealment (selection bias) |  | Quote: “subjects randomized to receive the medical assistance device were contacted by the local provider”  Comment: participants received the device or not, but randomization was clear selecting control and intervention participants. |
| Blinding of participants and personnel (performance bias) |  | Quote: ‘This study was not blinded. Subjects were obviously aware of having the device or not, and the coordinator obtaining outcome data was aware of the study purpose’  Comment: outcome measurement is not likely to be influenced by the lack of blinding |
| Blinding of outcome assessment (detection bias) (patient-reported outcomes) |  | Comment: outcome measurement is not likely to be influenced by the lack of blinding |
| Incomplete outcome data addressed (attrition bias) (Short-term outcomes  (2-6 weeks)) |  | Comment: Didn’t mention incomplete or missing data. Only one point post-measurement |
| Incomplete outcome data addressed (attrition bias) (Longer-term outcomes  (>6 weeks)) |  | Comment: Didn’t mention incomplete or missing data.  Only one point postmeasurement |
| Selective reporting (reporting bias) |  | Comment: lack of information about the control group, the project’s protocol is not available |
